# Abnormal Default System Functioning in Depression: Implications for Emotion Regulation

**DOI:** 10.3389/fpsyg.2016.00858

**Published:** 2016-06-10

**Authors:** Irene Messina, Francesca Bianco, Maria Cusinato, Vincenzo Calvo, Marco Sambin

**Affiliations:** Department of Philosophy, Sociology, Pedagogy and Applied Psychology, University of PadovaPadova, Italy

**Keywords:** depression, default system, emotion regulation, self, psychodynamic, psychotherapy, neuroimaging

## Abstract

Depression is widely seen as the result of difficulties in regulating emotions. Based on neuroimaging studies on voluntary emotion regulation, neurobiological models have focused on the concept of cognitive control, considering emotion regulation as a shift toward involving controlled processes associated with activation of the prefrontal and parietal executive areas, instead of responding automatically to emotional stimuli. According to such models, the weaker executive area activation observed in depressed patients is attributable to a lack of cognitive control over negative emotions. Going beyond the concept of cognitive control, psychodynamic models describe the development of individuals’ capacity to regulate their emotional states in mother-infant interactions during childhood, through the construction of the representation of the self, others, and relationships. In this mini-review, we link these psychodynamic models with recent findings regarding the abnormal functioning of the default system in depression. Consistently with psychodynamic models, psychological functions associated with the default system include self-related processing, semantic processes, and implicit forms of emotion regulation. The abnormal activation of the default system observed in depression may explain the dysfunctional aspects of emotion regulation typical of the condition, such as an exaggerated negative self-focus and rumination on self-esteem issues. We also discuss the clinical implications of these findings with reference to the therapeutic relationship as a key tool for revisiting impaired or distorted representations of the self and relational objects.

## Introduction

Depression is generally considered as the outcome of difficulties in regulating emotions (for reviews see Campbell-Sills and Barlow, 2007; Aldao et al., 2010). When dealing with their own emotions, depressed individuals tend to ruminate on (Nolen-Hoeksema, 2000; Garnefski and Kraaij, 2006), avoid or suppress thoughts and emotions associated with negative events (Wenzlaff and Rude, 2002; Campbell-Sills et al., 2006), whereas a reappraisal of the event from a different perspective (Gross, 2002; Webb et al., 2012) or a non-judgmental acceptance (Liverant et al., 2008; Kohl et al., 2012) would be more effective for the purpose of containing negative emotional activation and its physiological correlates. Given its importance for psychological wellbeing, emotion regulation is attributed a key role in psychological treatments for depression (Messina et al., 2013; Grecucci et al., 2015).

**FIGURE 1:**
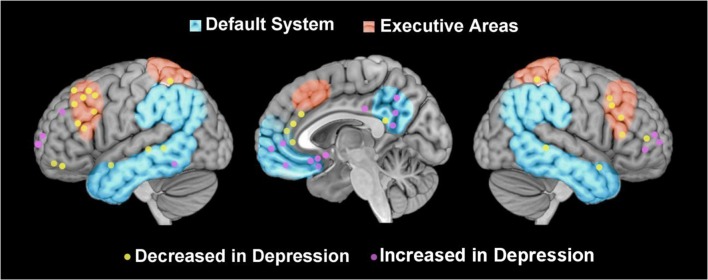
**Graphical representation of foci of perfusion reported in association with depression**. Graphical representation of foci of brain activity reported in studies that have compared depressed patients and healthy controls (in yellow decreased activations, in violet increased activations), located in executive areas [in red, retrieved from http://neurosynth.org/; (Yarkoni et al., 2011)], and default system (Raichle et al., 2001b).

Within the emerging field of affective neuroscience, the concept of voluntary emotion regulation has been widely used to explain the findings of functional neuroimaging studies conducted to elucidate the neural correlates of affective dysfunctions (for reviews, see Taylor and Liberzon, 2007; Menon, 2011). These studies have amply documented that individuals suffering from depression have a decreased activation of prefrontal cortex areas involved in executive control (including the dorsolateral prefrontal cortex – dlPFC –, and the dorsal anterior cingulate cortex – dACC), suggesting a weaker top–down control over their emotional reactivity deriving from the activation of limbic structures such as the amygdala (Drevets, 2001; Siegle et al., 2007). This interpretation is in line with fMRI studies concerning the neural correlates of emotion regulation that have revealed an increased activation of the prefrontal areas and a decreased activation of the amygdala in tasks involving the cognitive control of emotions by comparison with the spontaneous response to emotional stimuli (Ochsner and Gross, 2005; Buhle et al., 2014). Even if other authors have found that depressed individuals can display impaired emotion regulation despite preserved recruitment of dlPFC (Greening et al., 2014) and increased dlPFC recruitment during emotion regulation attempts (Johnstone et al., 2007), the emotion dysregulation seen in depression is consistently interpreted as a lack of cognitive control over emotional states (see **Figure 1**, the part in red, for a graphic representation of the executive areas involved in voluntary emotion regulation).

In addition to investigating brain activity in response to stimuli or during cognitive tasks, neuroscientists have become increasingly interested in the brain’s intrinsic activity in resting state. Studying resting state activity has led to the identification of the “default system,” a set of regions – including the ventral medial prefrontal cortex (vmPFC), the posterior cingulate cortex (pCC), the posterior parietal lobe, and the lateral, inferior and medial temporal cortices (see **Figure 1**, in blue) – that are usually activated at rest and deactivated during cognitively effortful tasks (Raichle et al., 2001a; Raichle and Snyder, 2007). Research on resting state activity has also been applied to investigating emotional disorders, comparing the resting-state brain signals of patients and healthy subjects (Broyd et al., 2009; Whitfield-Gabrieli and Ford, 2012). This field of research is generating new lines of inquiry for neurobiological models of emotion regulation and their application to interpreting the brain correlates of emotional disorders. In the present mini-review, we address this issue by examining the findings on the abnormal functioning of resting-state brain activity in depressed patients. We specifically consider these findings in the light of clinical concept, coming from psychodynamic tradition, which underscore the role of internal representations of the self and others in emotion dysregulation.

## Psychological Mechanisms: Internal Representations and Emotion Regulation

While the neurobiological models have conceptualized emotion regulation as a form of cognitive control, the psychodynamic tradition has concentrated more on investigating how individuals develop the ability to regulate their emotions in the course of their childhood, collecting evidence of the importance to emotion regulation of constructing a representation of the self and of relationships with others in their primary relationships. This interest is apparent in the works of Ferenczi (1933), Spitz (1945, 1965), Freud (1955), Bion (1959, 1967), Winnicott (1965), and Freud and Burlingham (1974), among others. These authors take the view that emotion regulation in infants depends on their caregivers’ contingent responses (on “good enough” parenting; Winnicott, 1965). For instance, Bion (1967) pointed out the caregivers’ important role in the infants’ acquisition of a comprehension and containment of their inner world through the transformation of the infant’s projected psychological experience in a “metabolized” form. Children can thus internalize the mother’s function and, with time, they learn to regulate their negative affective states.

Internalizing the mother’s function coincides with a self-building process described by Kernberg (1976) as the product of introjection and identification of the child’s significant relationship with the mother. Failure to achieve an interpersonal regulation in the mother-infant interaction may interfere with the construction of the self, and of its regulatory functions. Winnicott (1967) and Kohut (1971) used the term “weakened self” to describe the effects of a caregiver’s incongruous mirroring that oblige children to internalize distorted parental representations, which take the place of their subjective experience, preventing them from regulating that subjective experience. This is also implicit in Kernberg’s (1976) model, according to which a lack of differentiation between the internal representations of the self and others is an important factor contributing to emotional instability. With specific regard to depression, a mother’s inadequate mirroring function induces the child to internalize a rigid or even sadistic super-ego that implies a poor self-representation in which the subject feels helpless (Kohut, 1971, 1977; Kohut and Wolf, 1978).

The assumption that an individual’s internal representation of the self and others is important to emotion regulation has been the object of empirical investigation in the field of attachment theory (Bowlby, 1969, 1973, 1980). The attachment patterns characterizing the relationship between children and their caregivers can be seen as a particular form of dyadic regulation, in which the infant experiences the caregiver’s emotional availability (Cassidy, 1994). Within the primary relationship, adaptive emotion regulation strategies are learned by building “internal working models” (IWM; Bowlby, 1969), which are mental representations of the self and others based on the child’s daily experiences and expectations regarding a given caregiver’s response to attachment behavior. When the primary attachment figure is available and responsive to their needs, infants develop a sense of attachment security characterized by IWM that include positive beliefs regarding the self and others, the accessibility of others and their ability to alleviate distress, thus shaping affect regulation in the event of distress (Feeney and Cassidy, 2003; Fraley et al., 2006). But if infants have caregivers who are inconsistent and fail to respond adequately to their attachment needs, they develop a sense of attachment insecurity characterized by IWM that include negative representations of the self, low self-esteem and parenting self-esteem (Calvo and Bianco, 2015), difficulty in relying on others when their own emotional resources are insufficient, low dyadic adjustment, and loneliness (Shaver and Hazan, 1987; Kobak and Sceery, 1988; Sroufe, 2005). In recent years, insecure attachment has been quantitatively operationalized in terms of self-reported anxiety and avoidance in adult attachment relationships (Brennan et al., 1998), which imply two different maladaptive affect regulation strategies, called “secondary strategies” (Mikulincer et al., 2003; Shaver and Mikulincer, 2007): (i) strategies based on over-activation of the attachment system, including relational overdependence, desire to minimize physical, cognitive, and emotional distance from others; (ii) strategies based on deactivation of the attachment system, featuring the creation of emotional distance from others, avoidance of intimacy, and the suppression of negative feelings and memories. In the attachment literature, there is large evidence of depressive symptoms being associated with the chronic use of secondary emotion regulation strategies (Cole-Detke and Kobak, 1996; Rosenstein and Horowitz, 1996; Mickelson et al., 1997; Malik et al., 2015).

Recent contributions to this line of research have shown that the mirroring function of their attachment figures enables children to internalize the ability to think for themselves and perceive themselves as a thinking entity, a process called “mentalization” (Fonagy et al., 2007, 2011). In conditions of attachment security, children’s affective states are accurately (but not overwhelmingly) reflected back to them, whereas attachment insecurity and a lack of mirroring interfere with this mentalization process, giving rise to emotional disorders (Fonagy and Target, 1997; Bateman and Fonagy, 2004).

## Neural Mechanisms: The Default System in Emotion Regulation

While psychodynamic models of emotion regulation emphasize the importance of the internal representation of the self and others in explaining emotional disorders, neuroscience has concentrated more on emotion regulation as a form of cognitive control, neglecting the importance of the semantic representations on which controlling processes may act. Considering the default system in association with emotion dysregulation can compensate for this shortcoming.

### Default System Functions Linked to Emotion Regulation

The main feature of the default system is that it is activated during rest (Raichle et al., 2001a; Raichle and Snyder, 2007). It has been amply documented that, in the absence of a task that demands voluntary attention, the mind usually tends to wander (Mason et al., 2007). Activation of the default system is generally anti-correlated with activation of the executive areas (Fox et al., 2005), a situation reflecting the opposition between internal (default system) and external (executive functions) addressed attention.

In task-related studies, activation of the default system has been associated with cognitive tasks that include aspects of self-representation (Northoff et al., 2006) and self-projection, described as the capacity to project oneself mentally from the present into the past or future, but also from other people’s perspectives (Buckner and Carroll, 2007; Spreng et al., 2009). Self-projection underlies several processes that may be associated with emotional dysregulation, such as access to autobiographical memories (self-projection into the past) or future plans (self-projection into the future), but also with empathy and theory of mind (self-projection from others’ perspectives). In line with psychodynamic theory and research showing the importance of self-representation for emotion regulation, the evidence of the default system being activated in self-related processes suggests its involvement in emotion regulation. Intriguingly, the overlap between brain structures that are activated for self-representation and in theory of mind (Saxe et al., 2006) seems to confirm the psychodynamic view of a common source for the representations of the self and others constructed in infants’ primary relationships with their caregivers.

Another point in common with the psychodynamic models is the similarity between the default system and the semantic system, a set of regions activated by the retrieval and manipulation of conceptual knowledge gained from capturing regularities in the outside world (Binder et al., 2009). Semantic memory may include more complex representations of clinical relevance, however, such as those of relationships governing social interactions (Zahn et al., 2007), and representations of the self (Lou et al., 2004). In this setting, IWM can be seen as an example of representations that capture regularities in interpersonal relationships. Indeed, semantic areas are modulated by the exposition to attachment-related experimental stimuli, such as familiar faces (Gobbini et al., 2004) or of attachment eliciting pictures (Buchheim et al., 2006).

The activation of semantic areas is commonly described in voluntary emotion regulation studies, such as reappraisal (Buhle et al., 2014; Messina et al., 2015), but also in association with implicit forms of regulation in which executive functions are not involved, such as spontaneous avoidance (Benelli et al., 2012). Psychotherapeutic action has also been found associated with changes in semantic area activation (Messina et al., 2013). Going beyond the concept of emotion regulation as a form of cognitive control, recent recent contributions have begun to take into consideration the importance of semantic representation on which controlling processes may act (Blair and Mitchell, 2009; Buhle et al., 2014; Greening et al., 2014). According to recent models, semantic areas may play a key role in emotion regulation by conveying information about motivational priorities and facilitating processing of behaviorally relevant inputs (see Viviani, 2013, 2014; Messina et al., 2015).

### Abnormal Default System Activity in Depression

Due to the default system’s involvement in the self-related processing and semantic representation of repeated past experiences, an abnormal default system activity should be expected in depressed patients. Several studies have tried to clarify the specificity of resting-state brain functioning in depression, using positron emission tomography (PET) or the perfusion MRI technique known as arterial spin labeling (ASL). The methodological features of these studies are listed in **Table 1**. 

**Table 1:** List of studies on resting state in depression.

Studies	Experimental design	* N*	Patients details	Depression measure	Increased activation in depression	Decreased activation in depression	Positive correlation with depression	Negative correlation with depression
Drevets et al., 1992	PET 40 s Eyes closed and relax	13 DD 33 HC	>3 weeks medication washout	HDRS	L/R DMPFC L VLPFC L MPFC RL amygdala and other subcortical areas	L/R occipitalcortex R medialtemporalgyrus R/L medial caudate	Amygdala	L PFC
Mayberg et al., 1997	PET 40 min Eyes closed, ears uncovered, and resting state	18 DD 15 HC	Treatment with antidepressant medications	HAMD	–	R/L anterior cingulated R/L anterior insula R/L premotor cortex R/L DLPFC R/L IPL R/L inferiortemporal	–	–
Brody et al., 2001	PET 40 min Resting not specified	13 DD 24 HC	>4 weeks Medication washout	HAMD GDS	LR anterior inferior temporal	R/L DLPFC L/R dorsal caudate L/R thalamus	–	–
Saxena et al., 2001	PET 40 min Eyes and ears open	27 DD 17 HC	No antidepressant medications	HDRS GAS SADS-L	L/R Thalamus	L hippocampus	–	L hippocampus
Videbech et al., 2001	PET 15 min Relax and look at a tread-cross on a computer monitor	42 DD 47 HC	Treatment with antidepressant medications included	HDRS	R hippocampus L cerebellum L occipito-temporal gyrus	–	–	–
Kennedy et al., 2001	PET 35 min Resting not specified	13 DD	>2 weeks medication washout	HDRS	R sgACC	L DLPFC R ventral striatum	–	–
Dotson et al., 2009 (1)^∗^	PET 60 s Resting not specified	34 HC	Older males Treatment with antidepressant medications included	CES-D	–	–	–	Precentral gyrus VLPFC Temporal pole Cerebellum L IPL
Dotson et al., 2009 (2)^∗^	PET 60 s Resting not specified	26 HC	Older females Treatment with antidepressant medications included	CES-D	–	–	L/R IPL L Angular gyrus L middle occipital gyrus	cerebellum R VLPFC R inferior temporal gyrus R orbitofrontal gyrus R middle occipital gyrus insula R IPL R middle temporal gyrus
Monkul et al., 2012	PET 10 sessions of 50 s 10 Resting (eyes closed and rest)	20 DD 21 HC	No antidepressant medications	HDRS	PCC Caudate Parahippocampal gyrus	ACC DLPFC VLPFC	Thalamus Putamen	DLPFC VLPFC ACC
Roffman et al., 2014	PET 15 min Resting (not specified)	16 DD	Treatment with antidepressant medications	HAMD	–	–	R insula Precuneus	–
Lui et al., 2009 (1)^∗^	ASL Resting (eyes closed and relax)	24 DD 42 HC	Recurrent depressive disorder No antidepressant medications	HAMD CGISS	–	L DLPFC L occipital lobe R VLPFC L/R thalamus	–	
Lui et al., 2009 (2)^∗^	ASL Resting (eyes closed and relax)	37 DD 42 HC	Non recurrent depressive disorder No antidepressant medications	HAMD CGISS	R/L occipital lobe R amygdala and other subcortical areas PCC/precuneus	L DLPFC	–	–
Duhameau et al., 2010	ASL 4 min Resting (not specified)	6 DD 6 HC	Drug resistant and chronic depression Treatment with antidepressant medications	HDRS	R sgACC L sgACC Corpus callosum L DMPFC L ACC L amygdala and other subcortical areas	–	–	–
Orosz et al., 2012	ASL 12 min Resting	22 DD 22 HC	Treatment with antidepressant medications	BDI HAMD MADRS	–	–	–	PCC IPL

*^∗^We reported separately different comparison published in the same article. DD, Depressive Disorder patients; HC, healthy controls; BDI, Beck Depression Inventory (Beck et al., 1961); HAMD, Hamilton Depression Rating Scale (Hamilton, 1960); MADRS, Montgomery-Asberg Depression Rating (Montgomery et al., 1985); GDS, Geriatric Depression Scale (Brink et al., 1982); CGISS, Clinical Global Impression of Severity scale (Guy, 1976). (Hamilton, 1960; Beck et al., 1961; Guy, 1976; Brink et al., 1982; Montgomery et al., 1985; Drevets et al., 1992; Mayberg et al., 1997; Brody et al., 2001; Kennedy et al., 2001; Saxena et al., 2001; Videbech et al., 2001; Dotson et al., 2009; Lui et al., 2009; Duhameau et al., 2010; Monkul et al., 2012; Orosz et al., 2012; Roffman et al., 2014)*.

Both PET and ASL studies have produced evidence of less activity in the frontal executive areas of the brains of depressed patients compared with healthy controls, especially in the dlPFC (Mayberg et al., 1997; Kennedy et al., 2001; Lui et al., 2009; Monkul et al., 2012), but also in the dACC (Mayberg et al., 1997; Monkul et al., 2012) (see **Figure 1**). A greater activation has also often been reported in subcortical areas, including the amygdala, in depressed patients (Drevets, 2001; Lui et al., 2009; Duhameau et al., 2010). These results are consistent with the neurobiological model of emotion regulation that postulates a weaker cognitive control of emotions in depression.

In addition to the prefrontal-subcortical network of voluntary emotion regulation, studies on resting-state brain activity have reported foci of increased activation located in the anterior part of the default system, extending from the subgenual ACC to the anterior portion of the ACC or the vmPFC (Drevets, 2001; Kennedy et al., 2001; Duhameau et al., 2010), and also to posterior portions of the default system such as the PCC and precuneus (Lui et al., 2009; Monkul et al., 2012). Judging from evidence of how the default system functions, these results may suggest a greater negative self-focus and attention to internal thoughts in depressed patients than in controls, coinciding with the rumination processes characteristic of depression. Consistently with this hypothesis, increased default system activation has been associated to individual differences in maladaptive rumination (Hamilton et al., 2011). In psychodynamic terms, this rumination can be described as an “internal dialog”: if they have failed to mentalize some emotional states (which are often relational needs that have been not mirrored in primary relationships), individuals use forms of emotion regulation based on suppression or avoidance (“defense mechanisms” in psychodynamic terms–Freud, 1967), instead of mentalizing their emotional responses flexibly to suit their relational needs.

### Clinical Implications: Therapy and Changing Internal Representations

If representations of the self and others are constructed within a close relationship, these internal representations may also be changed by means of a close relationship. The therapeutic relationship can be seen as a relational context in which patients act out their attachment patterns and can make changes to the internal representations of the self and others (Lyddon and Sherry, 2001; Dales, 2008). Researchers have provided strong empirical evidence of the outcome of psychotherapy being mediated by the quality of the therapeutic relationship (Horvath, 2002), with includes the therapist’s empathy (Burns and Nolen-Hoeksema, 1992; Elliott et al., 2011) and ability to engage the patient in a therapeutic alliance (Martin et al., 2000). The importance of therapeutic relationships is a factor spanning all psychotherapy techniques (Meares et al., 2005; Norcross and Lambert, 2011).

In psychodynamic psychotherapy, the work on internal representations of the self and others is also expressed in specific techniques that aim to explore how patients’ internal models influence their relationship with the therapist in order to arrive at cognitive but also emotional insights on the influence of their primary relationships on their current relationships. The most popular example is transference interpretation (Freud, 1912). An evolution of transference interpretation is the “triangle of insight” (Menninger, 1958), widely used in brief psychodynamic psychotherapy (Malan, 1976; Davanloo, 1980), but with potential applications also in other psychotherapeutic approaches. Using the triangle of insight, therapists and patients observe together how past experiences (the first vertex of the triangle) influence current life experiences (the second vertex), and are manifested in the here-and-now of the therapeutic relationship (the third vertex). In the following transcript of psychotherapy intervention, we provide an example of the triangle of insight in Intensive Transactional Analysis Therapy:

T: *What’s the problem?* (current problem)
P: *Well… I have a lot of anxiety when I have to speak in front of lots of people*
T: *Even now?* (Therapist links patient’s current life experience with the here-and-now of the therapeutic relationship)
P: *Yes*
T: *Okay! Where is this anxiety?*
P: *Here* (points to chest). *My heart is racing and I am sweating*
T: *Your heart is racing…and…is something else that is happening?*
P: *Eh… I have confusion in my head*
T: *Do you have confusion now?*
P: (nods)
T: *Can you explain what this confusion is…* (Therapist continues the exploration of third vertex of triangle of insight and patient’s emotion regulation)

## Conclusion, Outstanding Questions, and Future Directions

The core idea emerging from the present mini-review is that the default system is abnormally activated in patients with depression, consistently with the observation of negative self-focus and rumination in such patients. In line with clinical models coming from psychodynamic theory, these difficulties in emotion regulation can be seen as associated with the existence of rigid, negative internal representations of the self and others. Considering such processes in neurobiological models of emotion dysregulation helps us to build bridges between the theories behind clinical psychology and neuroscience. Several gaps remain, however, in this attempt at integration. One question remaining to be answered concerns how processes of cognitive control and internal representation of the self and others interact in engendering rumination and avoidance instead of adaptive strategies for regulating emotions. With this in mind, future studies should clarify how individual differences in default system activation, and this system’s correlations with other brain networks are associated with the complexity and flexibility of internal representation.

## Author Contributions

IM conceived the review and wrote the sections “Introduction,” “Neural mechanisms,” and “Conclusion” of the manuscript. FB wrote “Psychological mechanisms” and MC wrote “Clinical implications”. VC and MS supervised the manuscript. All authors listed, have made substantial, direct and intellectual contribution to the work, and approved it for publication.

## Conflict of Interest Statement

The authors declare that the research was conducted in the absence of any commercial or financial relationships that could be construed as a potential conflict of interest.
